# Delayed administration of allogeneic cardiac stem cell therapy for acute myocardial infarction could ameliorate adverse remodeling: experimental study in swine

**DOI:** 10.1186/s12967-015-0512-2

**Published:** 2015-05-12

**Authors:** Veronica Crisostomo, Claudia Baez-Diaz, Juan Maestre, Monica Garcia-Lindo, Fei Sun, Javier G. Casado, Rebeca Blazquez, Jose L. Abad, Itziar Palacios, Luis Rodriguez-Borlado, Francisco M. Sanchez-Margallo

**Affiliations:** Jesús Usón Minimally Invasive Surgery Centre, Carretera N-521, km 41.8, 10071 Cáceres, Spain; Coretherapix, Santiago Grisolía, n° 2 Parque Científico de Madrid, 28760 Tres Cantos, Madrid Spain

**Keywords:** Cardiac stem cells, Myocardial infarction, Cardiac remodeling, Allogeneic, Experimental

## Abstract

**Background:**

The optimal timing of cardiac stem cells administration is still unclear. We assessed the safety of same-day and delayed (one week) delivery and the possible influence of the timing on the therapeutic outcomes of allogeneic porcine cardiac stem cells administration after acute myocardial infarction in a closed-chest ischemia-reperfusion model.

**Methods:**

Female swine surviving 90 min occlusion of the mid left anterior descending coronary artery received an intracoronary injection of 25x10^6^ porcine cardiac stem cells either two hours (n = 5, D0) or 7 days (n = 6, D7) after reperfusion. Controls received intracoronary injection of vehicle on day 7 (n = 6, CON). Safety was defined in terms of absence of major cardiac events, changes to the ECG during injection, post-administration coronary flow assessed using the TIMI scale and cardiac troponin I determination after the intervention. Cardiac Magnetic Resonance was performed for morphological and functional assessment prior to infarction, before injection (D7 and CON groups only), at one and 10 weeks. Samples were taken from the infarct and transition areas for pathological examination.

**Results:**

No major adverse cardiac events were seen during injection in any group. Animals receiving the therapy on the same day of infarction (D0 group) showed mild transient ST changes during injection (n = 4) and, in one case, slightly compromised coronary flow (TIMI 2). Cardiac function parameters and infarct sizes were not significantly different between groups, with a trend towards higher ejection fraction in the treated groups. Ventricular volumes indexed to body surface area increased over time in control animals, and decreased by the end of the study in animals receiving the therapy, significantly so when comparing End Diastolic Volume between CON and D7 groups (CON: 121.70 ml/m^2^ ± 26.09 ml/m^2^, D7: 98.71 ml/m^2^ ± 8.30 ml/m^2^, p = 0.037). The treated groups showed less organization of the collagenous scar, and a significantly (p = 0.019) higher amount of larger, more mature vessels at the infarct border.

**Conclusions:**

The intracoronary injection of 25x10^6^ allogeneic cardiac stem cells is generally safe, both early and 7 days after experimental infarction, and alleviates myocardial dysfunction, with a greater limitation of left ventricular remodeling when performed at one week.

**Electronic supplementary material:**

The online version of this article (doi:10.1186/s12967-015-0512-2) contains supplementary material, which is available to authorized users.

## Background

Cardiovascular diseases remain a major cause of death and disability, with coronary heart disease being responsible for 20 % of all deaths in Europe [1]. Adult stem cells are emerging as a therapeutic option, with a modest improvement in cardiac function after cell transplantation [2, 3]. In recent years, cardiac stem cells (CSCs) have been proved in numerous preclinical studies to improve left ventricular function and attenuate remodeling after myocardial infarction [4-12]. Based on these experimental works, human trials have been initiated. Promising results have been reported from the use of autologous cardiac cells in the setting of heart failure, either after coronary artery bypass grafting [13, 14] (SCIPIO trial, NCT00474461) or after coronary stenting [15, 16] (CADUCEUS trial, NCT00893360).

However, the autologous approach presents obvious drawbacks in that the time needed for cell expansion conditions the time frame for treatment. Allogeneic cells emerge as a promising option, and several studies using this strategy have been reported [7, 17-23]. An allogeneic approach offers the possibility of administering cells very early after the ischemic event, as an “off-the-shelf” product that, at the same time, can be subjected to higher quality controls than autologous products [24, 25]. The earliest possible administration has been advocated [2, 24, 26, 27]. Data from clinical trials using bone marrow cells, however, seem to support a best effect of these cells when administered after the 4^th^ day post-infarction [28]. To date, allogeneic CSCs have been tested in swine 2-3 weeks [5] and immediately [22, 23] after infarction. The optimal timing of CSCs administration is still unclear. The myocardial substrate immediately after infarction may be detrimental for cell engraftment, due to an early rise in reactive oxygen species and inflammatory cytokines. Conversely, once the inflammatory phase is over, scar formation and adverse remodeling are under way, and therefore the effect of the cells may be limited [29], although there are also studies reporting beneficial effects at subacute and chronic settings [30, 31].

We undertook this study to assess the safety of early delivery and the possible influence of the timing of allogeneic porcine CSCs (pCSCs) delivery after acute myocardial infarction (AMI) on cardiac function in a closed-chest swine model of reperfused myocardial infarction.

## Methods

### Experimental protocol

The study protocol was approved by the Institutional Animal Care and Use Committee, and it complied fully with the Directive 2010/63/EU of the European Parliament on the protection of animals used for scientific purposes.

**Fig. 1:**
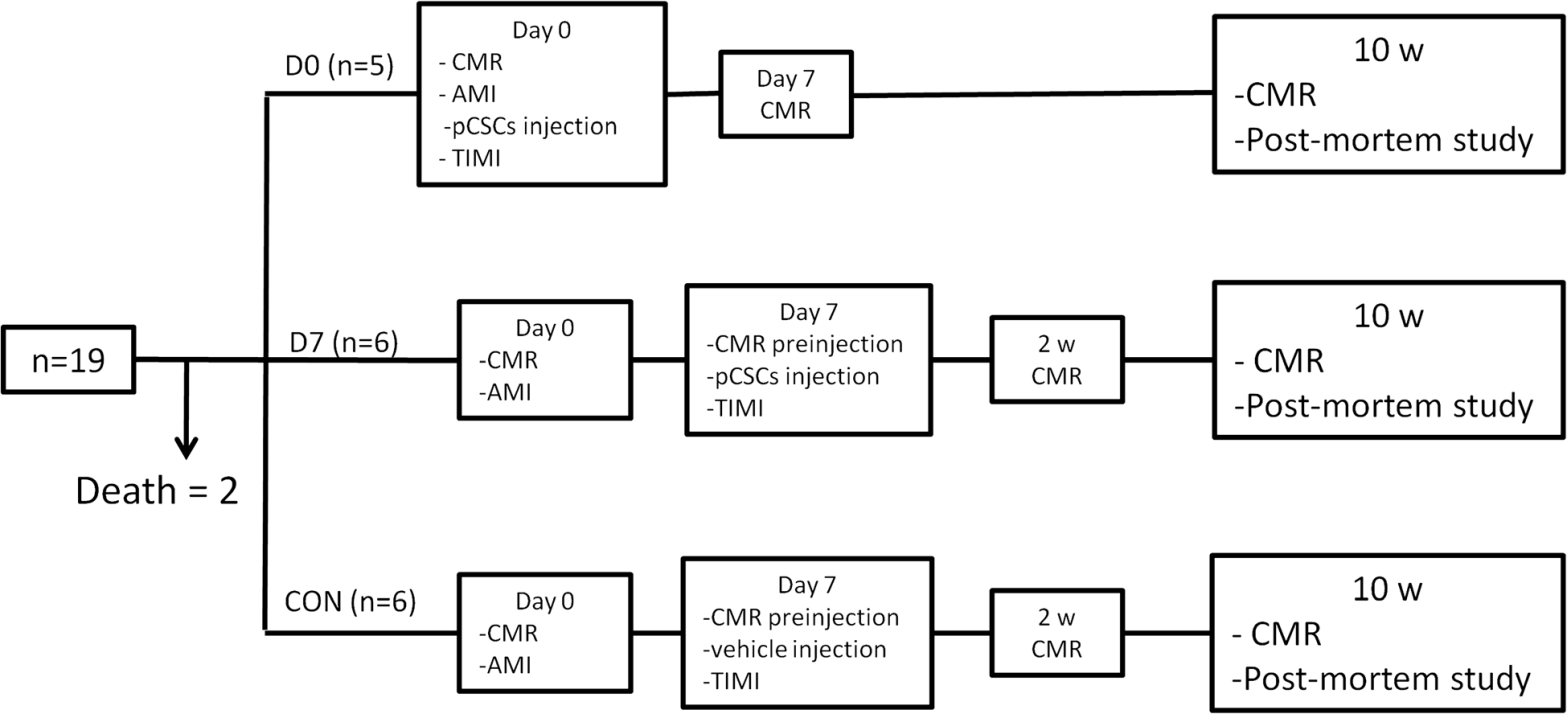
Study design. n = number of pigs. AMI = acute myocardial infarction. pCSC = porcine cardiac stem cells. TIMI = Thrombolysis in myocardial infarction. CMR = cardiac magnetic resonance

The study flow chart is presented in Fig. 1. Female domestic swine weighing 30-35 kg were used for this study (n = 19). Prior to inclusion in the protocol, all animals underwent a complete physical examination and serum biochemical analyses. Study animals received 400 mg oral amiodarone from 5 days prior to infarct induction to 3 days after it, and 500 mg aspirin and 300 mg clopidogrel 24 h before model creation. The antithrombotic medications continued through the study until euthanasia was performed (500 mg aspirin and 75 mg clopidogrel). All the procedures were performed under general anaesthesia.

### Anaesthesia and monitoring protocols

After being fasted for 24 h, animals were premedicated by the intramuscular injection of ketamine (20 mg/kg). 10 min after premedication, access to an ear’s marginal vein was obtained and anaesthesia was induced with intravenous (IV) 1 % propofol (3 mg/kg). Endotracheal intubation was performed using cuffed endotracheal tubes (sizes 6.5 -9, depending on the animal’s weight).

For cardiac magnetic resonance (cMR) examinations, anaesthetic maintenance was performed with a continuous propofol infusion (8-12 mg/kg/h). Animals were connected to a MR-compatible ventilator (TransPAC T200, Smiths Industries Medical Systems, UK) and mechanical pressure controlled ventilation was established with a FiO2 of 0.5 to assure normocapnia.

During infarct induction and pCSCs administration, anaesthesia was maintained using a continuous IV infusion of a combination of 1 % propofol (10-12 mg/kg/h) and remifentanyl (15-18 μg/kg/h). Endotracheal tubes were connected to a semi closed circular anaesthetic circuit attached to a ventilator (Leon Plus, Heinen & Löwenstein, Bad Ems, Germany) with a fresh gas flow rate of 1 L/min (0.4/0.6 mixture of oxygen and air). Controlled ventilation was established with a tidal volume of 6-8 mL/kg to obtain normocapnia (with a CO_2_ pressure of 35-40 mmHg).

Systemic heparin was injected IV (150 IU/kg) 5 min prior to percutaneous sheath placement.

Anaesthetic monitoring included cardiovascular and hemodynamic parameters such as: heart rate, electrocardiography, pulse-oxymetry and invasive arterial blood pressure. Ventilatory parameters registered were: respiratory rate, oxymetry, airways pressure, inspired and end-tidal CO_2_ concentration.

Once the follow-up was completed, animals were euthanized by a lethal dose of potassium chloride (1-2 mmol/kg) while under deep anaesthesia, as recommended by the American Veterinary Medical Association (AVMA Guidelines for the Euthanasia of Animals: 2013 Edition. Available at: https://www.avma.org/kb/policies/documents/euthanasia.pdf).

### Infarct induction protocol

For infarct creation, animals were fixed at the table in the dorsal decubitus with caudal extension of the hind limbs, and the groin area was prepared for access. Under sterile conditions, a right femoral arterial access was established using the Seldinger technique and a 7 Fr introducer sheath (Terumo, Inc. Tokyo, Japan) was placed percutaneously into the femoral artery. Under fluoroscopic guidance (Philips Mobile Digital Angiographic System-BV Pulsera, Philips Medical Systems, Best, The Netherlands) a 6 Fr hockey stick guiding catheter (Mach 1 ®, Boston Scientific Corporation, Natick, MA, USA) was introduced and placed at the origin of the left coronary artery. 150mg of IV amiodarone in 5 % glucose saline were administered slowly prior to left anterior descending coronary artery (LAD) engagement with the guiding catheter. As soon as the guiding catheter was placed at the origin of the left coronary artery, 150 μg of nitroglycerin were administered through it to prevent coronary spasm. Coronary angiograms were then obtained in the 40° left anterior oblique (LAO) projection to better demonstrate the length of the LAD, and a 0.014” guidewire (Hi-torque. Abbott Vascular, Santa Clara, CA, USA) was advanced inside the LAD. After measuring the diameter of the LAD immediately below the origin of the first diagonal branch, a coronary bare metal stent (Apolo. Iberhospitex SA, Barcelona, Spain) of appropriate diameter was advanced to this location. Before occluding the artery, a bolus of 2 % lidocaine (1mg/kg) was administered and the balloon was then inflated. Correct occlusion was assessed by contrast injection through the guiding catheter immediately after balloon inflation and before deflation. The occlusion was maintained for 90 min. In the event of the animals developing ventricular fibrillation during the occlusion, manual chest compressions and 200 J biphasic defibrillation shocks (Zoll M series biphasic 200J, Zoll Medical Corporation, Massachusetts, USA), and pharmacological therapy when needed were used to revert them. After balloon deflation and removal, a post procedural coronary angiogram was obtained to assess coronary patency and flow, which was scored following the Thrombolysis In Myocardial Infarction (TIMI) grade flow. Animals were kept under anaesthesia with lidocaine infusion for another hour, and were then recovered and sent back to the animal housing facility for postoperative observation. Subjects allocated to injection on day 0 were kept under close supervision and lidocaine infusion for another hour, before performing the pCSCs injection as described below. In all cases, postoperative analgesia was obtained with 10 μg/kg/12h of IM buprenorphine during the first 24h. A fentanyl transdermic release patch (25 μg/h) was used to assure correct analgesia in the immediate postoperative period. Prophylactic antibiotics were administered in all cases for 5 days after infarct induction (ceftiofur hydrochloride).

### pCSCs preparation

Porcine CSCs were obtained from male Large White pigs. After mechanical and enzymatic digestion of heart tissue samples, a cellular suspension was obtained and CD45 positive cells immunodepleted. CSCs were immunoselected from cell suspension using anti cKit antibodies. Isolated cells were seeded in a culture plate and in vitro expanded using culture medium with 10 % Foetal Bovine Serum (FBS) and growth factors to obtain a Working Cell Bank (WCB). Cells at WCB were frozen in DMEM medium with 10 % of pig serum and 10 % of DMSO at 10x10^6^ cells /ml and stored in a liquid nitrogen tank. The pCSCs were characterized by flow cytometry, real time quantitative PCR (qPCR) and ELISA. For cell administration, vials were thawed in a 37°C bath and the cell suspension mixed with 25 ml of saline buffer with 5 % of bovine serum albumin. Cells were centrifuged and the supernatant removed. The cell pellet was resuspended in phosphate saline buffer (PBS) with 5 % of BSA (bovine serum albumin) and passed through a 40 μm cell strainer. Cell concentration and viability was tested using trypan blue exclusion and cell concentration adjusted at 2.1x10^6^ cell /mL for administration using PBS-5 % BSA.

### Group allocation and pCSCs administration

Surviving animals were sequentially allocated to the control group (CON, n = 6), pCSCs injection on day 0 (D0, n = 5) or pCSC injection on day 7 (D7, n = 6).

In the D0 group, the administration was performed 2 h after reperfusion, to allow for stabilization of the animal. In the CON and D7 groups, administration was performed 7 days after infarct induction, immediately after acquiring a cMR study. Access to the LAD was established using the same protocol described for infarct creation, and a 21 Fr microcatheter (Microferret infusion catheter, Cook Medical. Bloomington, IN, USA) was placed at the level of the coronary stent. The total volume to be administered was divided in two 6mL syringes, and administered in two injection cycles separated by a 3 min rest period. Each half dose was injected manually over 3 min. Once the stated volume was infused, we waited for 5 min before obtaining a coronary angiogram to assess coronary TIMI grade flow again. The femoral sheath was then removed and haemostasia of the puncture site achieved by manual compression.

Blood samples were taken for cardiac troponin I (cTpnI) assay before infarction and 24h after reperfusion (all groups) and also before and 24h after pCSCs/vehicle administration (CON and D7 groups) (AQT90 Flex, Radiometer Iberica SL, Madrid, Spain).

### MR examinations

Cardiac Magnetic Resonance studies were performed for morphological and functional assessment before injection (CON and D7 groups), one and 10 weeks after pCSCs/vehicle infusion.

In all cases, animals were placed inside the MR system (Intera 1.5 T, Philips Medical Systems. Best, The Netherlands) in the sternal decubitus. Retrospective cardiac triggering was used. A 4 elements phase array coil was placed around the animals’ chest. Images were acquired in the intrinsic cardiac planes: short axis, vertical long axis and horizontal long axis views. For measurement of left ventricular function and mass breath hold gradient echo cine images were obtained over the entire left ventricle (LV). Typical parameters used were: slice thickness: 8 mm, no gap, Field of view (FOV): 320 x 320 x 80, matrix: 192x192, flip angle: 60°, repetition time/echo time (TR/TE): 4.4/2.2. For infarct size measurements, short axis images were acquired 5 to 15 min after the injection of 0.2 mmol/kg of a gadolinium-based contrast agent (Gadobutrol. Gadovist 1.1 mmol/l, Bayer Schering Pharma AG, Berlin, Germany) using a breath-hold 3D gradient-echo inversion-recovery sequence. Inversion time was chosen for each sequence using a Look-Locker sequence and selecting the time that yielded the best nulling of the myocardial signal, which typically ranged from 150 to 190 ms. Imaging parameters used for the delayed enhancement images were slice thickness: 8 mm, no gap, FOV: 330 x 330 x 50, matrix: 224x200, flip angle: 15°, TR/TE: 4.9/1.67. MR images were analyzed by a researcher blinded to the group allocation using a commercially available software (Extended MR WorkSpace 2.6.3.2. Philips Medical Systems. Best. The Netherlands.). For these analyses, endocardial and epicardial borders were manually delineated in end diastolic and end systolic short axis views, in all slices, and the following LV functional parameters were calculated: end diastolic volume (EDV), end systolic volume (ESV) and ejection fraction (EF). The thickness of the infarcted (septum) and healthy lateral free wall were also measured in end diastolic short axis views. Similarly, infarct size calculations were performed in the delayed enhancement images by manually defining the normal and infarcted myocardium with computer assistance to obtain the percentage of infarcted left ventricle. Central dark zones within the area of hyperenhancement were included. All determinations were performed by an investigator blinded to the group allocation.

In order to perform a robust comparison and avoid the influence of the animal’s growth on the results, volume data were indexed by Body Surface Area (BSA), using the weight-based formula described by Kelley [32].

### End of study and post-mortem examinations

Animals were euthanized 10 weeks after infarction. On the allotted euthanasia day, immediately after the corresponding cMR study, a coronary angiogram was obtained via a percutaneous access to a femoral artery for TIMI flow grade scoring and euthanasia was performed as described above. Prior to hearts explantation, pericardial fluid (PF) samples were collected for TNF-alpha and active TGF-beta levels quantification. Cytokines were measured using quantitative ELISA kits for porcine TNF-alpha (R&D systems) and active TGF-beta (BioLoegend, San Diego, CA). PFs were centrifuged for 5 min at 450 x g, passed through a 0.22 μm filter and frozen at -20°C until assayed. PFs were thawed at room temperature and ELISA was performed according to manufacturer´s instructions. ELISA plates were read at 450 nm using the ELISA Reader (Synergy MX BioTek). Results were expressed as picograms of cytokine per ml of PF. Explanted hearts were photographed to document macroscopic infarct distribution, cut into serial slices and placed in a 1 % solution of 2,5,3-triphenyl tetrazolium chloride (TTC) in phosphate buffer at 37°C for 10 min.

Samples were taken from the infarct and transition areas for pathological examination, embedded in paraffin, sliced into 5 μm thick sections and stained with haematoxylin-eosin and Masson’s trichromic. As previously described [21], the density and size of blood vessels present at the infarct border was determined from five randomly chosen areas of each sample. A scoring system comprising from 0 (absent or normal) to 4 (severe) was used to score the presence of inflammatory infiltrate, fibrosis, necrosis, calcification or teratoma formation.

For the detection of male-derived pCSCs in female hearts, Y-chromosome PCR was carried out from tissue samples obtained from the transition area in all hearts. DNA extraction was performed with TRI Reagent (Sigma, St. Louis, MO, USA) according to the manufacturer's instructions. The detection of male cells in a female recipient was carried out by PCR using the Taq DNA Polymerase (Invitrogen, Carlsbad, Ca). Amplification consisted in 40 cycles of 30 s at 94°C for melting, 30 s at 55°C for annealing and one min at 72°C for amplification. The primers 5´-ACAGAGGGCCTATTCATCTCAG-3´ (forward) and 5´-CTTAATGGCTAATCACGGGAAC-3´ (reverse) were designed to allow the amplification of Y-chromosome specific sequences (NCBI Reference Sequence: NC_010462.2).

### Statistical analysis

Data are presented as means ± standard deviations. Data were checked for normality using the Shapiro Wilks test. Differences between groups were identified and compared using the Kruskal-Wallis and Mann-Whitney U tests. Values of p < 0.05 were considered significant. Calculations were performed using the SPSS 18.0 statistical package for Windows (SPSS Inc, Chicago, Ill).

## Results

A total of 19 female pigs were used for this study. One of the animals died during infarct induction, before group allocation, and a second animal belonging to D0 died during the stabilizing period, prior to pCSCs administration. Infarction was successfully induced in all surviving animals, as demonstrated by an increase in Troponin I values to over our system detection limit (>25 μg/L) in all animals 24 h after model creation. No further animal deaths, of cardiac or any other origin, were seen in this study.

**Fig. 2:**
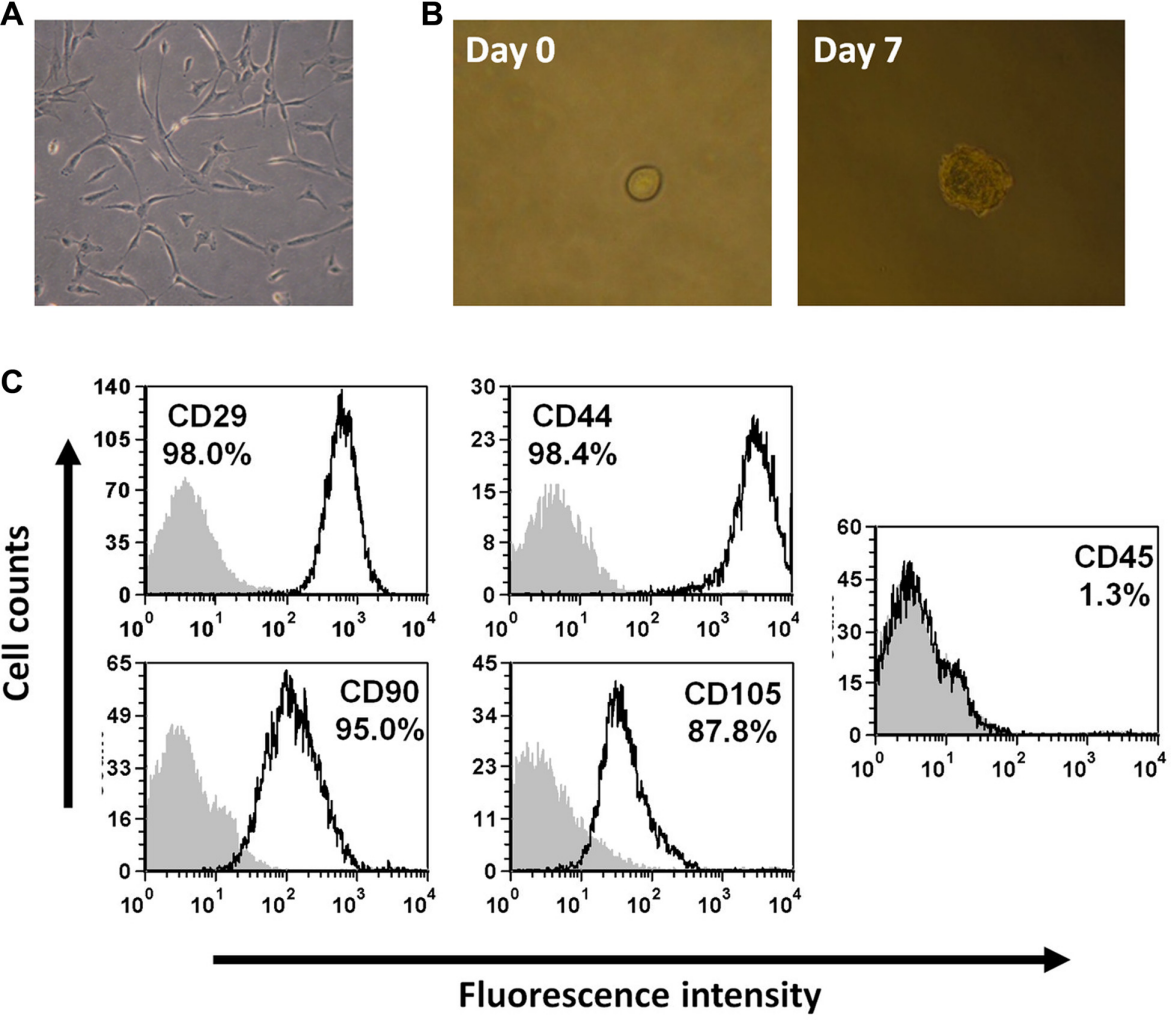
Porcine CSCs characterization. **a** Morphology of porcine CSCs in a primary cell culture. **b** Porcine CSCs are able to proliferate in non-adherent conditions forming cardiospheres after 7 days in suspension. **c** Cell surface expression analysis by flow cytometry. Expression of CD90, CD44, CD29, CD105 and CD45 is shown (empty histogram) and the number of positive cells is indicated (%). Grey filled area represents isotype control

As indicated above, pig CSCs were phenotypically characterized by flow cytometry, qPCR and ELISA. Pig cells were positive for CD29, CD44, CD90, CD105, and GATA4 and negative for CD45 (Fig. 2) and secrete IGF-1, TGF-β and CCL2 factors. In addition, isolated pig cells had clonogenic capabilities and were able to differentiate in vitro to smooth muscle, endothelial cells and cardiomyocytes (see Additional files 1 and 2). The phenotypical characterization indicated that in vitro expanded porcine cells were equivalent to previously described human CSCs [33]. Post-thaw viability of cryopreserved pCSCs was >95 % and no aggregates were present.

Injection was successful in all animals at both 0 and 7 days after AMI, in absence of major adverse cardiac events (MACEs). Slight ST-segment elevations were seen in 4 animals from D0 during the injection procedure, but the ST wave spontaneously returned to pre-injection values once the administration was completed in all cases. No changes in the ECG were seen during pCSCs/vehicle administration in any CON or D7 animal.

Post injection coronary flow was scored as TIMI 3 in all animals but one belonging to the D0 group, in which TIMI 2 flow was demonstrated after pCSCs administration.

A slight increase in Troponin I levels was seen in D7 and CON 24 h after therapy administration (Table 1), but this increase was not significantly different between groups (Mann Whitney U-test, p = 0.46). Troponin I in D0 animals was elevated beyond the higher detection limit of the laboratory, (>25 μg/L). However, in these animals, this time point represented also the 24 h after infarction measurement.

**Table 1:** Cardiac Tpn values (μg/l) measured during the study

GROUP	Baseline (pre infarction)	24 hours post-infarction	1 week post-infarction	24 hours post-cells
CON	0.060 ± 0.052	>25	0.034 ± 0.029	0.327 ± 0.275
D7	0.022 ± 0.024	>25	0.021 ± 0.009	0.542 ± 0.985
D0	0.049 ± 0.058	>25	n/a	>25

Data presented as mean ± standard deviation

**Fig. 3:**
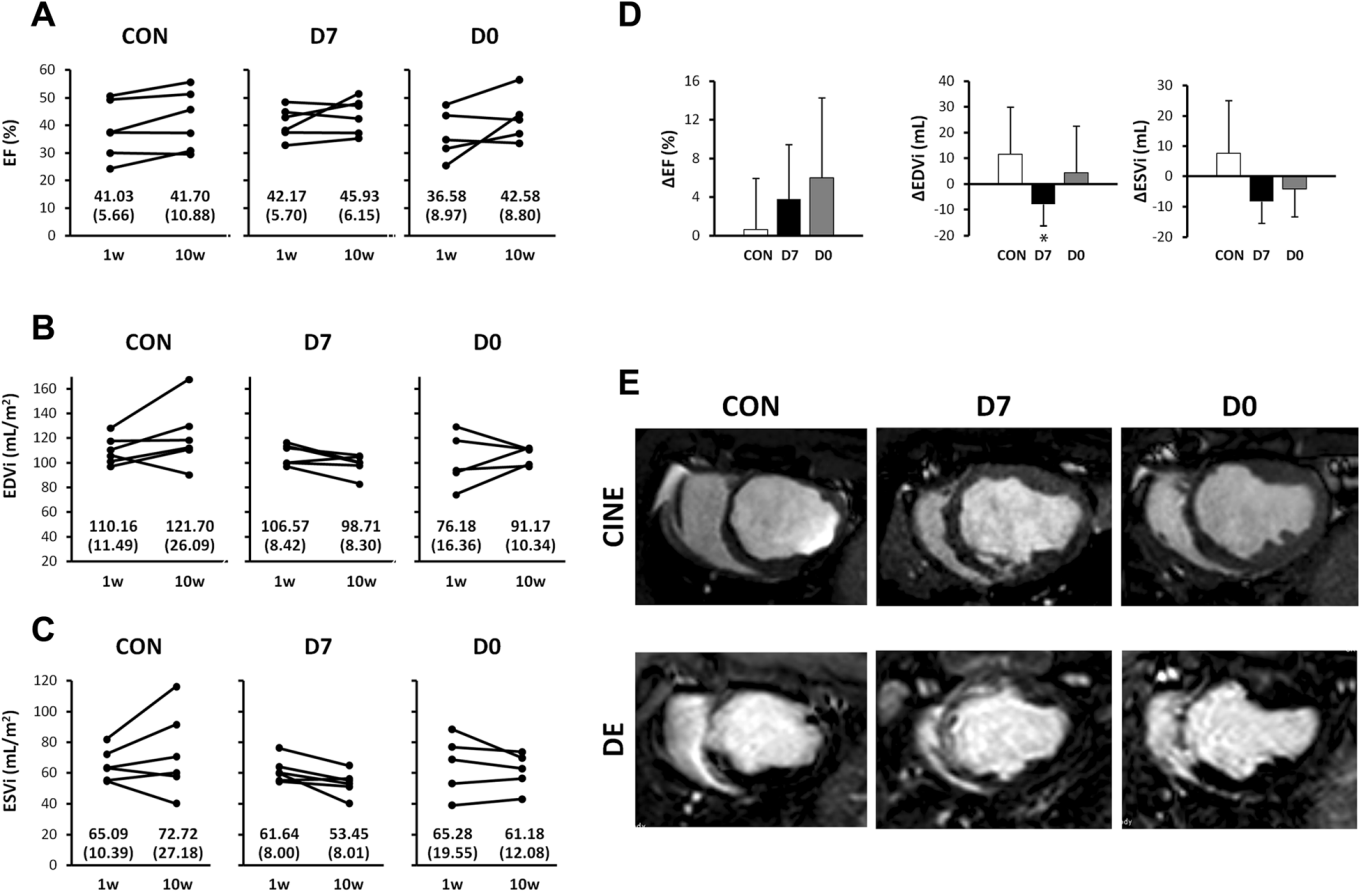
Changes over time in cardiac function parameters. Cardiac function was measured with cardiac magnetic resonance imaging. EF = Left ventricular ejection fraction. EDVi = Indexed end diastolic volume. ESVi = Indexed end systolic volume. DE = delayed enhancement. Panels **a** to **c** show the evolution of these parameters in individual animals over time. **d**: Changes between groups. * Denotes statistical significance compared to CON (p < 0.05). **E**: Cardiac MR images obtained at 10 weeks after infarct induction in animals receiving intracoronary vehicle injection on day 7 (CON), or injection of 25x10^6^ pCSCs either two hours (D0) or 7 days (D7) after reperfusion

CMR-derived cardiac function data are presented in Table 2 and Fig. 3. No differences were seen between groups in baseline functional data. Intergroup comparisons revealed no significant differences in EF or infarct sizes at any time point between study groups. Indexed ventricular volumes after 10 weeks were greater in the control group, but this difference only reached statistical significance in EDVi between CON and D7 on week 10 (p = 0.037, Mann Whitney *U* test). The evolution of these parameters within each group over time is shown in Fig. 3. In absence of significant differences, EF increased slightly in both treated groups, while in the control group it remained stable. Similarly, EDVi decreased in D7 from 1 week after injection to the end of the study (N.S), while it increased slightly in D0 animals. ESVi, however, decreased over time in both treated groups. A clear trends towards increased indexed volumes was seen in CON animals (EDVi was 98.71mL/m^2^ ± 8.3mL/m^2^, 106.04mL/m^2^ ± 7.26mL/m^2^ and 121.70mL/m^2^ ± 26.09mL/m^2^ and ESVi was 53.45mL/m^2^ ± 8.01mL/m^2^, 61.18mL/m^2^ ± 12.08mL/m^2^ and 72.72mL/m^2^ ± 27.18mL/m^2^, respectively in D7, D0 and CON). Septal thickness showed a trend towards a greater thinning in the CON group, followed byD7 and D0 animals, while free LV lateral wall thickness increased slightly in all cases, in absence of significant intergroup differences (Table 3).

**Table 2:** Cardiac parameters calculated from Magnetic Resonance exams performed through the study

	CON group				D7 group				D0 group		
	Baseline (preinfarction)	Day 0 preinjection	1 week	10 weeks	Baseline (preinfarction)	Day 0 preinjection	1 week	10 weeks	Baseline (preinfarction)	1 week	10 weeks
Weight (kg)	32.5 ± 1.87	33.8 ± 1.33	33.0 ± 1.67	39.17 ± 2.71	30.67 ± 1.97	31.00 ± 3.80	30.33 ± 2.73	41.33 ± 5.01	31.60 ± 2.07	34.60 ± 1.67	42.60 ± 4.98
EF (%)	51.57 ± 7.48	38.22 ± 10.4	41.03 ± 5.66	41.70 ± 10.88	51.08 ± 7.68	41.07 ± 7.50	42.17 ± 5.70	45.93 ± 6.15	54.24 ± 8.63	36.58 ± 8.97	42.58 ± 8.80
EDVi (mL/m^2^)	82.61 ± 18.36	106.24 ± 10.96	110.16 ± 11.49	121.70 ± 26.09*	79.18 ± 13.20	97.86 ± 13.00	106.57 ± 8.42	98.71 ± 8.30*	79.95 ± 7.59	101.65 ± 22.02	106.04 ± 7.26
ESVi (mL/m^2^)	39.97 ± 9.47	66.1 ± 15.74	65.09 ± 10.39	72.72 ± 27.18	38.92 ± 9.65	57.60 ± 9.85	61.64 ± 8.00	53.45 ± 8.01	36.94 ± 9.58	65.28 ± 19.55	61.18 ± 12.08
% Infarct	n/a	17.20 ± 5.54	11.33 ± 2.94	7.50 ± 2.07	n/a	16.33 ± 3.61	13.33 ± 4.88	10.00 ± 4.29	n/a	17.00 ± 3.46	9.40 ± 2.79
Infarct mass (g)	n/a	13.28 ± 4.96	7.46 ± 1.92	5.25 ± 1.32	n/a	10.49 ± 2.65	7.95 ± 2.36	6.20 ± 2.45	n/a	12.16 ± 3.88	7.03 ± 2.29

Data presented as mean ± standard deviation. EF: Ejection fraction. EDVi: End diastolic volume indexed to body surface area. ESVi: End systolic volume indexed to body surface area. n/a: Not applicable. * P < 0.05 (Mann Whitney *U* test. Intergroup comparisons at each time point)

**Table 3:** Left ventricular wall thickness (mm) over time

	Baseline (preinfarction)		Day 0 preinjection		1 week		10 weeks	
	Septum	Lateral wall	Septum	Lateral wall	Septum	Lateral wall	Septum	Lateral wall
CON	7.68 ± 1.25	7.28 ± 0.59	6.86 ± 1.81	7.85 ± 0.40	5.24 ± 2.23	8.08 ± 1.67	2.98 ± 0.28	7.97 ± 0.73
D7	6.18 ± 1.08	6.68 ± 1.13	6.40 ± 1.07	7.41 ± 0.76	4.79 ± 1.30	7.21 ± 0.64	3.69 ± 0.76	7.68 ± 0.41
D0	6.82 ± 0.76	6.99 ± 0.67	n/a	n/a	7.22 ± 0.93	6.94 ± 0.60	4.66 ± 1.61	8.56 ± 0.76

Data presented as mean ± standard deviation

An area of hyperenhancement comprising the mid anterior, anteroseptal and apical septal left ventricle was visualized on the follow up cMR examinations. The size of the infarct, measured in percentage, decreased over time in all groups (Table 2, Fig. 3).

**Fig. 4:**
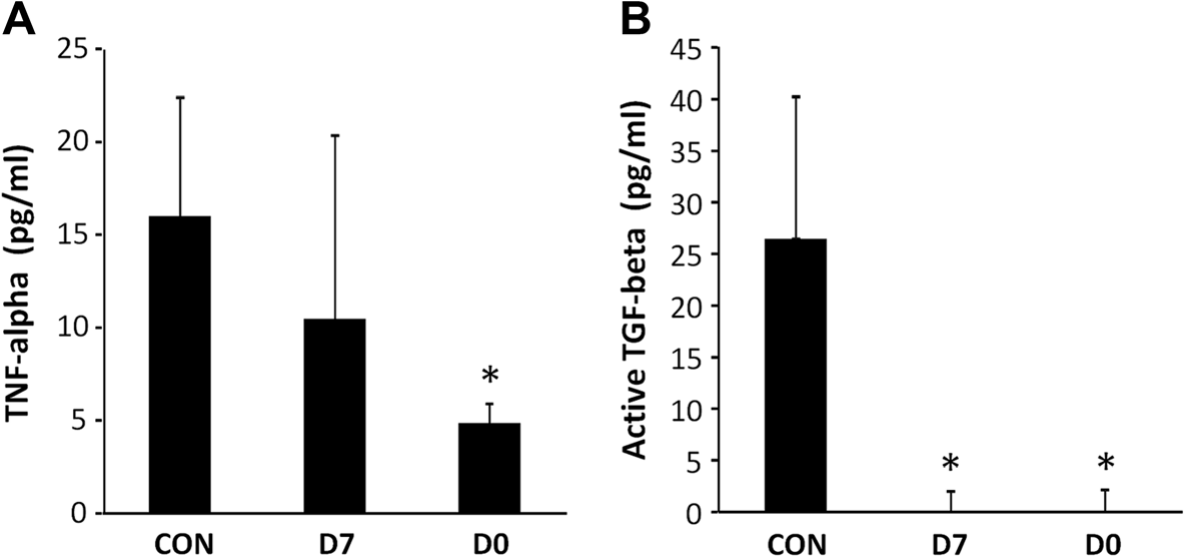
Cytokines levels in pericardial fluid. Cytokines levels were measured in the filtered pericardial fluid using quantitative ELISA kits for porcine TNF-alpha and active TGF-beta. Absorbance was read at 450 nm and cytokines levels (expressed in pg/ml) were extrapolated from a calibration curve. The graphs represent mean values ± standard deviation of four independently performed experiments (n = 4) for TNF-alpha (**a**) and active TGF-beta (**b**) determinations. The results were analyzed through a Student’s *t*-test for variables with parametric distribution, comparing each measure with the control group (*p < .05)

TNF-alpha and active TGF-beta levels were quantified by ELISA in PF collected upon euthanasia. As shown in Fig. 4, TNF-alpha levels were significantly lower in the groups of animals where pCSCs were injected 2 h after infarction, compared to not injected animals. However, there were not significant differences between control and animals injected one week after the infarction (Fig. 4A). On the other hand, the levels of active TGF-beta in PF significantly decreased when pCSCs were administered, both in D7 and D0 groups, compared to control group (Fig. 4B).

**Fig. 5:**
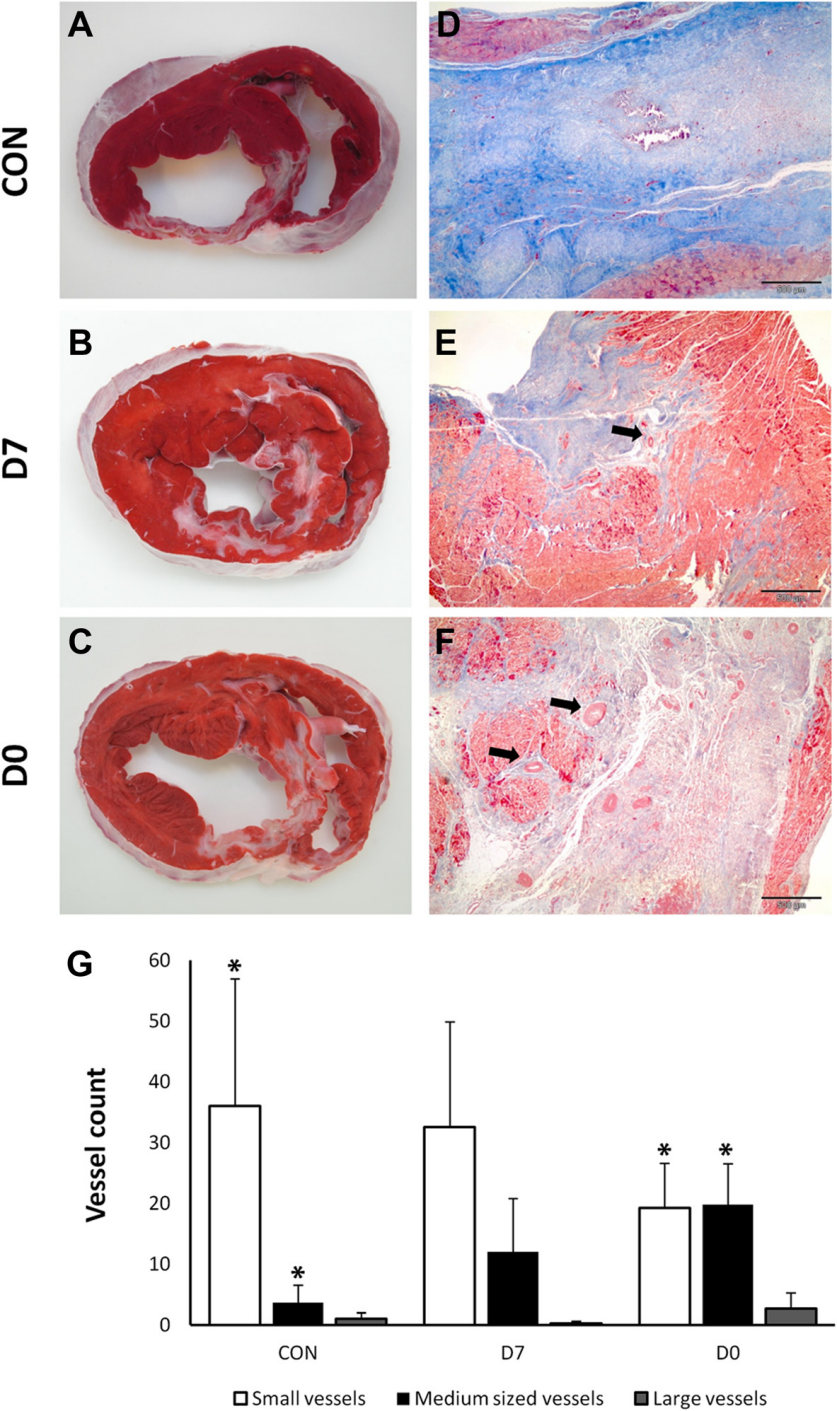
Typical histological and macroscopical appearance of the infarcts. Panels **a** through **c**: TTC staining shows the extension of infarcted tissue in the different groups. Panels **d** through **F** Massons trichromic evidences increased collagen in the control animals, while some medium to large size vessels can be seen in treated animals (arrows). The bar represents 500 μm. **G**. Distribution of vessels’ sizes as determined at the infarct border. * denotes statistical significance (p < 0.05)

Upon euthanasia, anteroseptal transmural fibrous scars of varied extension were seen in all hearts, with a certain degree of thinning of the left ventricular wall evident in all cases. However, thinning was greater in animals belonging to the CON group, which, on TTC staining, exhibited a clear area of non viable myocardium. In the treated groups, thinning was less, and there was some viable tissue surrounding and infiltrating the scars (Fig. 5).

On light microscopy, these scars were fibrotic with different degrees of organization and some scattered inflammatory infiltrate. Histopathological findings are summarized in Table 4. CON samples exhibited the highest degree of dense fibrosis (severe), with almost complete replacement of myocardiocytes by a mature fibrotic scar, while the treated groups showed less organization of the collagen content (considered moderate in D0 and slight in D7), interspersed with variably sized clusters of viable myocardiocytes. Overall, there were no major differences between groups in terms of inflammation (mild to moderate), presence of necrosis or calcification (mostly absent, except in one animal belonging to CON and one belonging to D0). No evidence of teratoma formation was seen in any case. No differences between groups were seen in the total amount of vessels. However, regarding the sizes of these vessels, Differences were seen between groups using the non parametric Kruskal Wallis test (p = 0.019). Post-hoc comparisons showed that the treated groups had a higher amount of mid and large caliber (and therefore more mature) vessels compared to CON, significantly so in the case of D0 (p = 0.008) (Fig. 5).

**Table 4:** Summary of histopathological findings

	CON (n = 6)	D7 (n = 6)	D0 (n = 5)
Inflammatory infiltrate	1.5	1	2
Fibrosis	4	1	3
Necrosis	2	1	2
Calcification	0	0	0
Teratoma	0	0	0

Data presented as median scores. 0: Absent, 1: slight, 2: mild, 3 moderate and 4: severe

**Fig. 6:**
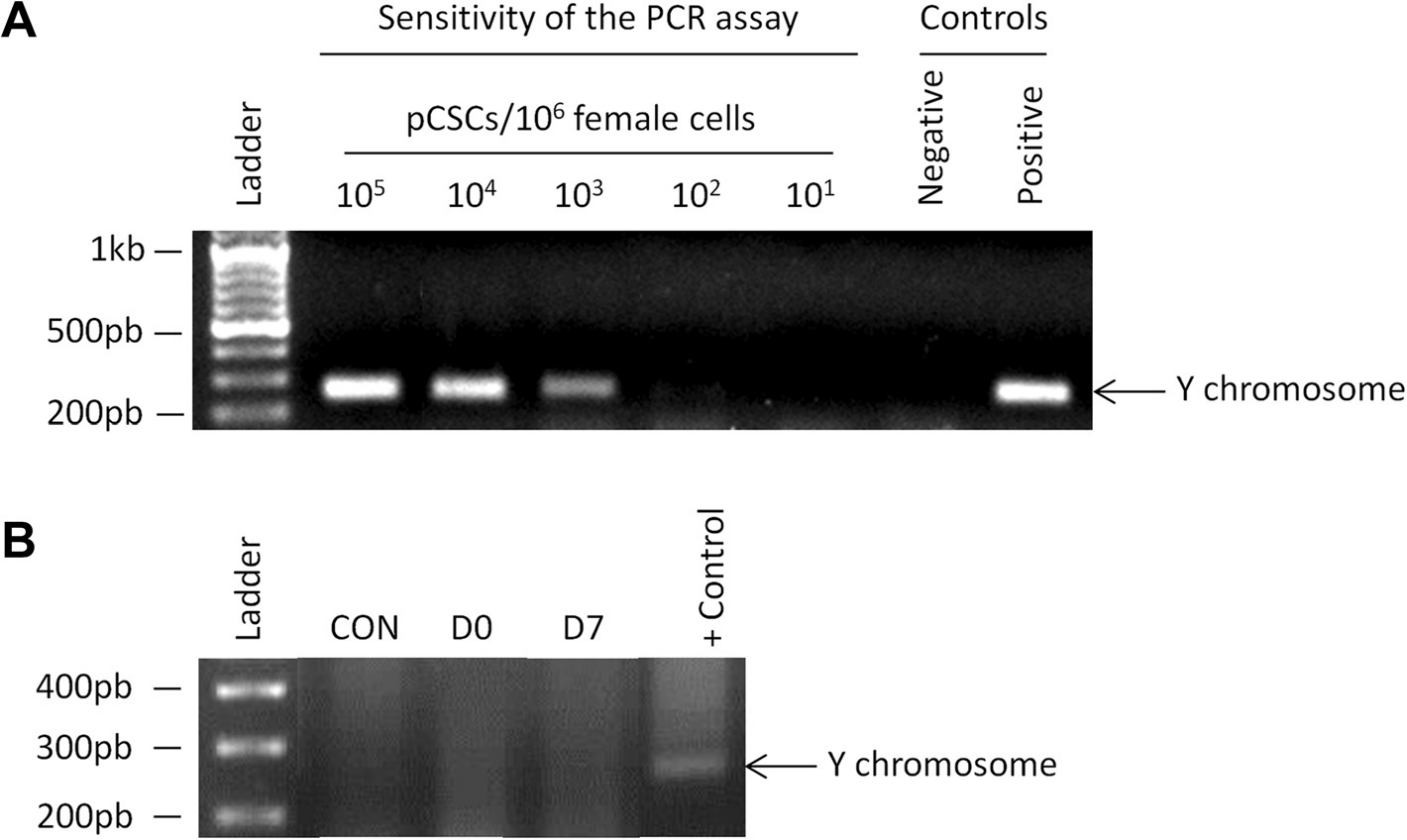
Y chromosome detection of intracoronary delivered pCSCs. **a** In order to determine the sensitivity of PCR amplification, pCSCs from a male donor were mixed with 10^6^ pCSCs from a female donor at the indicated ratios. Genomic DNA was extracted and subjected to PCR amplification using Y chromosome specific primers. PCR allowed the detection of male cells with a sensitivity of 100-1000 cells per 10^6^ female cells. **b** Female pigs were intracoronary injected with male-derived pCSCs. After euthanasia, heart tissue samples were collected for PCR analysis. A representative PCR for each group is shown. As negative and positive controls, the genomic DNA from female and male pCSCs cells were amplified

In order to determine the detection limit of the technique for Y chromosome detection into the female recipients, preliminary experiments were performed. Samples with 10^1^, 10^2^, 10^3^, 10^4^ and 10^5^ male cells were mixed together with one million female cells and amplified by PCR. This technique showed a detection limit of 100-1000 male cells per 10^6^ female cells (Fig. 6A). For the detection of transferred cells, the DNA from different tissues was isolated and Y chromosome amplified by PCR. However, as shown in Fig. 6B, male pCSCs were not detected in any of the samples ten weeks post-administration.

## Discussion

The beneficial effect of CSC administration in the setting of myocardial infarction has been demonstrated in numerous preclinical studies [5-8, 10-12] and preliminary clinical data [13-16] have reported unprecedented good results using heart derived cellular products. SCIPIO investigators reported a 12.3 % increase in LVEF at one year [13], whereas the patients enrolled in the CADUCEUS trial did not show any changes in this parameter compared to the control group after one year, despite demonstrating a 11.1 % reduction in scar size (compared to unchanged scar size in control patients) and an increase in viable myocardium by 22.6g in treated patients versus 1.8g in controls [15]. In the present study, we focus on the timing of allogeneic CSCs administration via the intracoronary route in a closed-chest model of MI. While some results with heart derived products administered immediately after experimental infarction have been reported in abstract form [22, 23], to the best of our knowledge, no comparative timing study using CSCs has been performed. Our findings following the intracoronary injection of 25x10^6^ confirm the safety of this approach, both early and 7 days after experimental AMI, with no MACEs during administration or later cardiac deaths seen in any animal. In terms of efficacy, our functional results suggest a better performance of the pCSCs when administered one week after the ischemic insult, while pathological examination points to enhanced angiogenesis at both time points, especially so in the animals receiving pCSCs early after infarction.

The use of clinically compatible methods and techniques ensures comparability with other works and a rapid translation of preclinical studies [3]. In this study we not only used a widely accepted animal model of MI, such as the closed chest reperfused balloon occlusion porcine model, but also clinically compatible methods for cell infusion and to study the evolution of heart function. Swine present many similarities to the human heart and there is an increasing amount of preclinical works performed in pigs available for comparison, that can help interpreting and putting the results in context [3].

Intracoronary administration is, from a clinical point of view, the most practical method for cell delivery to the heart, as it is widely available worldwide, less invasive than other administration routes and the cells can be administered to the entire myocardium at risk [4, 10]. Concerns have been raised, however, regarding the safety of this approach, as several studies using cells from other [19, 24, 34] and cardiac [6] origin have reported instances of no reflow phenomena or increased troponin, thus dictating a limitation on the dose [34], the use of an optimized infusate [6] or infusion rate [24]. Nonetheless, there are many reports using this route in absence of complications for different cell types [5, 10, 13, 14, 20, 35]. To ensure the safety of our approach, we performed a pilot study with intracoronary injection of pCSCs in healthy swine, and we did not find any evidence of microinfarction or cardiac toxicity by either cMR or pathological assessment [36]. As previously reported [10, 24], the size of the cells used in this study, which ranged between 12-13 μm, may confer a clear advantage for their intracoronary administration over other stem cells currently in use.

In spite of its accuracy and reproducibility making it the preferred modality for inter-study comparisons [3], cardiac MR is not frequently used in preclinical studies [34]. Considering that cell therapy may cause changes in vessel density or architecture (increased wash out or decreased extravasation of contrast), Malliaras et al. recently studied the kinetics of gadolinium within the infarcted swine heart with or without cell therapy, finding no differences between groups and thus confirming the usefulness of this technique for monitoring regenerative efficacy [5].

In order to be able to test the administration of the therapy immediately upon reperfusion, we have used allogeneic cells, as previously performed by others using cardiac-derived cell products [5, 7, 11, 22, 23]. The advantages of using allogeneic cells have recently been highlighted by different groups [25, 30, 37, 38]. Most of the work performed with allogeneic cells in the heart has used MSCs. Clinically, the POSEIDON study, while not powered to show efficacy, reported similar safety profiles between allogeneic and autologous cell sources [30]. Preclinically, a recent meta-analysis including 82 papers dealing with large animal studies of cell therapy in ischemic heart disease showed that both cell types yielded similar increases in LVEF and decreases in EDV compared to placebo [38]. Since only one of those studies dealing with allogeneic cells included in the meta-analysis used immunosuppression, the authors support that allogeneic cell therapy can be performed without immunosuppression with positive results on functional cardiac parameters. Accordingly, we used no immunosuppressive therapy in this study, an approach that has been proved safe in other works [5, 7, 30, 38, 39], and that may open the door to a real off-the-shelf treatment option for cardiac diseases [37].

Nonetheless, and looking towards clinical translation, the allogeneic rejection risk of CSCs equivalent to those we have used has been recently described. In this paper, the authors demonstrated that hCSCs shift their signaling capacities within the allogeneic setting towards signals that promote the development, maintenance, and functioning of anti-inflammatory response [33]. Moreover, a thorough characterization of the immunologic properties of cardiac derived stem cells for allogeneic use has been performed [7]. In that study, the baseline immunophenotype of the cells supported an allogeneic use: CDCs were found to express MHC class I antigens, which confer protection against natural killer cell-mediated deletion, while no MHC class II antigens were identified, allowing the cells to escape direct recognition from CD4+ T helper cells.

In this paper, we also aimed to compare the TNF-alpha and TGF-beta levels between different study groups. Although other authors have determined these cytokines in serum or plasma [40], here we have quantified them in the pericardial fluid at 10 weeks post-infarction. We consider that the measurements of these cytokines in the pericardial fluid may better reflect the immune status in the infarcted hearts. The first cytokine we measured was TNF-alpha which is produced (at least in part) by the myocardium [41] and has been extensively described in different animal models of cardiovascular failure [42]. As expected, our measurements in the pericardial fluid showed high levels of TNF-alpha in the CON group (infarcted animals without cells). This result is in agreement with a previous report using rats as animal models of acute myocardial infarction [43] and clinical data from infarcted patients [44]. In these papers, rats and humans showed higher levels of TNF-alpha after long-term follow-up. The relevance of our results lies in the significant decrease of TNF-alpha observed in those infarcted animals which received an intracoronary administration of cardiac stem cells. Our results appear to be different from prior reports [40], where TNF-alpha levels were similar following cellular transplantation. However, there are several differences between the experimental procedures that could account for this difference in results: Firstly, we measured cytokines in PF rather than in serum. Secondly, the administered stem cells are different (bone marrow-derived stem cells in Schuleri´s report and cardiac stem cells in our work). And thirdly, the administration routes are also different (epicardial versus intracoronary administration). These differences may suggest that, either intracoronary administration and/or the cardiac stem cells may favor the decrease of TNF-alpha levels.

Regarding TGF-beta, our study has been only focused in the quantification of active TGF-beta in the pericardial fluid. Similarly to other studies, our results demonstrated higher levels of this cytokine in the pericardial fluid of untreated infarcted animals [45]. Surprisingly, TGF-beta was significantly lower in those infarcted which received an intracoronary administration of cardiac stem cells, either immediately or 7 days after infarction. These results are difficult to interpret because of the pleiotropic role of TGF-beta [46]. However, as TGF-beta levels were quantified in the active form, here we hypothesize that some of the TGF-beta activators (MMP-2, MMP-9, Thrombospondin-1, reactive oxygen species, acidic environment and others) could be down modulated in those animals which received the intracoronary administration of cardiac stem cells. Although this hypothesis should be further demonstrated, previous reports have demonstrated that intracoronary administration of adult stem cells induces the downregulation of metalloproteinases in myocardium (MMP2 and MMP9) after myocardial infarct [47].

Finally, it is important to note that, the analysis of only two cytokines would never provide an accurate description of immunological status after myocardial infarction. Indeed, a deeper analysis may include a wider range of cytokines, chemokines and growth factors. In any case, according to our modest and reduced analysis and taking into account the abundant literature demonstrating the immunomodulatory properties of these cells, we consider that intracoronary administration of pCSCs may provide an anti-inflammatory environment after myocardial infarction. However, 10 weeks post-administration pCSCs were not detected in any of the samples. We hypothesize that the anti-inflammatory environment could be mediated by the paracrine stimulation of endogenous regenerative mechanisms which include the recruitment and expansion of endogenous stem cells with strong immunomodulatory properties [48], although this hypothesis was not tested in our study.

Cellular therapy may work via the *de novo* formation of myocytes and vascular structures, the activation and growth of resident progenitor cells via a paracrine effect mediated by the implanted cells, or a protective effect of the cells (and their released factors) on the myocardium at risk [49]. These three mechanisms are not mutually exclusive, and different groups have published evidences of all three [4, 12, 26, 39]. The relative roles of these different mechanisms of action in cardiac derived cell products have been studied in immunocompromised mice receiving Cardiosphere-derived cells (CDCs) [50]. This group injected human CDCs in the peri-infarct area of SCID mice after permanent coronary ligation, in order to assess the direct and paracrine contributions of cardiac derived cell therapy to the regeneration obtained after administration in an infarcted heart and quantify the relative contributions of each mechanism to the beneficial effects observed after therapy. Since they use human cells in a mice model, they were able to track the paracrine factors secreted by human cells at one and three weeks after cell administration, as well as identify cells of human origin newly integrated into the cardiac capillaries and muscles. On the one hand, no original (luciferase labelled) CDCs could be detected at three weeks. On the other hand, and despite an overall doubling of capillary density, only 9.6 ± 2.7 % of the total capillaries were of human origin, and in the muscular component, only 11.8 ± 4.5 of detected myosin heavy chain was human in origin. They conclude that the major contributors to the mechanism of regeneration are paracrine effects, exerted both on the myocytes and on endogenous stem cells.

We therefore hypothesize that the administration of pCSCs in our model puts in motion a paracrine system that activates survival pathways on the cells at risk and activates the endogenous stem cell compartment [48]. Once these events are triggered, the presence of the cells is not needed to maintain the benefit, as demonstrated in prior studies with heart derived products, where no exogenous cell was found on the myocardium 3 weeks past the administration [11, 50, 51]. Similarly, we did not find any evidence of Y-chromosome in samples from our female hearts transplanted with male pCSCs 10 weeks after administration. However, allogeneic transplantations have been reported to exert sustained beneficial effects on infarcted hearts’ structure and function that persist over time, up to 6 months post treatment [7]. This short term permanence of the cells in the myocardium and the apparent long term benefit conferred regardless of it is another argument in favour of the absence of immunosuppressive therapy.

The timing of cell delivery may be critical, and there are arguments for both early and deferred administration in the setting of acute myocardial infarction. The optimal time is probably defined by the equilibrium between the positive and negative influence of the various cytokines present at the myocardium at this time [29]. Most experimental studies administer the therapy either immediately or at around one week after MI [3]. Those groups advocating the earliest possible administration of the cells after infarction [2, 4, 26, 27] support this approach based on the experimental data documenting a beneficial effect on the cells at risk by the paracrine action of the exogenous cells. Moreover, the administration of IGF-1 and HGF directly into the infarct related artery in experimental infarctions in swine improved cardiomyocyte survival, decreased collagen deposition and enhanced angiogenesis at 60 days after AMI, thus resulting in decreased scar formation and improved ventricular function [4]. Immediately after reperfusion, the inflammatory cardiac milieu may adversely affect the cells function and survival. In order to be able to exert a beneficial influence, the cells first need to survive the acute stage. There have been different ways to address this, such as the promotion of cell survival, for example by the concurrent administration of simvastatin [52] or by activation of Akt, a serine-threonine kinase [18], or by the transplantation of high doses of cells. Richardson et al reported a recent study comparing early and late administration of two doses of MSCs in infarcted rats, describing that, while all four groups receiving MSCs exhibited some improvement compared to control animals, this improvement was greater in the early high dose (2x10^6^) and lower in the early low dose (10^6^) administration group, while no difference between cell doses was seen on animals receiving MSCs at one week [17].

Other authors prefer to defer the administration until after this detrimental inflammatory microenvironment has subsided, to avoid the widespread death of the transplanted cells. Clinically, a benefit has been observed when BMCs are administered 5 days or later after reperfusion, with no effect of the cells when infused within 4 days of the ischemic event [28]. A recent meta-analysis of large animal studies showed a trend towards better results when cells injection was performed over 1 week after infarction [3].

In our case, there were benefits at both time points, but the apparent advantages of delayed administration both in terms of safety profile and of limitation of cardiac remodeling point to delayed administration being more effective. Despite being the most widely used parameter to gauge cardiac function, ejection fraction can be influenced by several confounding variables, and is highly dependent on afterload and preload, as well as cardiac rhythm, rate and ventricular shape [16]. On the other hand, left ventricular dilation after infarction has been considered the major identifiable risk factor for subsequent cardiac death [53]. ESV, in particular, was described in White’s classical work as the major determinant of survival after MI. The dilatation of the ventricular cavity can be attributed, among numerous other factors, to infarct expansion and ventricular wall thinning secondary to the scar formation and the replacement of viable, contractile myocytes for stiff collagen fibers, thus increasing wall stress in a process that ultimately may lead to heart failure. In this study conducted with a limited sample size we did not observe significant changes in EF, although the trend towards EF recovery seen in both treated groups was absent in the CON group. More promising are the changes seen in ventricular volumes, as depicted in Fig. 3. ESVi increased by almost 10 % in the CON animals by week 10, while both treated groups showed a decrease in this parameter, (by 8 % in D7 animals and 7 % in D0 animals), thus pointing to a recovery of systolic function in the treated groups. In the case of D7, EDVi decreases also from 1 week after treatment to the end of the study, when this difference is significant compared to CON (98.71 ± 8.30 mL/m^2^ in D7 versus 121.70 ± 26.09 mL/m^2^ in CON). Neovascularisation, on the other hand, may have been more advanced in the immediate administration group. Angiogenesis is considered determinant for improved cardiac function in response to cell therapy. There was no increase in the amount of vessels in treated animals compared to controls, but the infarct border in treated animals presented larger, more mature vessels, more so in the D0 group, suggesting an association of more mature vascularisation with the early administration of pCSCs in this experimental setting. Other studies have reported similar results, with the amount of vessels being similar between treated and control animals, but with cell therapy using MSCs causing an increase in these vessels’ size [21].

Infarct size decreased similarly in all three groups over time. However, microscopical evaluation of the infarcted areas revealed the existence of viable myocyte bundles within the scar in both treated groups, whereas Control animals exhibited a dense collagenous scar, as seen in other studies [4, 5, 11, 12]. The existence of these interspersed viable muscle bundles may determine a decreased stiffness of the infarcted wall, and therefore explain the improved functional results in the treated groups, in absence of differences in infarct size.

### Limitations

We recognize that the experimental setting can never be fully representative of the clinical scenario, as we use healthy, juvenile pigs without any co-morbidities, while cardiac patients are generally middle-aged to elderly people suffering from associated cardiovascular problems and risk factors, such as hypertension, atherosclerosis, and diabetes. Moreover, the animals used in the present study are still growing. In order to minimize the influence of weight gain on our interpretation of the results, as previously reported by others [35], volumes have been indexed to Body Surface Area. Clinical trials using CDCs have reported myocardial regeneration, in the form of increased grams of viable myocardium tissue over time [15]. Considering the pig’s growth, this kind of effect could not be assessed in our work. In order to minimize mortality due to infarct creation, we have administered amiodarone prior to model induction, an strategy that has allowed us to greatly decrease mortality in prior studies [54]. However, it is important to note that, although we consider it unlikely, this may have masked any arrhythmogenic effects during pCSCs administration

The experimental design defining CSCs administration on the same day of infarction in group D0 precluded the acquisition of a baseline (preinjection) CMR in this group. Since the MR acquisitions need to be gated for the cardiac cycle, and it is compromised in that premature ventricular complexes and tachycardia are common after balloon deflation, the quality of the images that can be obtained in this setting is poor, and often not of diagnostic quality. The first MR study in this group was obtained one week after CSCs administration, and we cannot know whether at this time point there was any effect of the cells. For this reason, comparisons between groups have focused on the 10 weeks time point. This limitation could have been circumvented by the use of other imaging techniques that are not so reliant on the cardiac cycle, such as echocardiography, but this technique in swine is especially difficult due to the animals’ thoracic wall configuration, and thus extremely operator-dependant.

This study was conducted using a small sample size, which could account for the general lack of statistically significant differences between groups. However, preclinical trials in large animal models rarely use a high experimental number [3], probably due to a combination of economical, logistical and ethical issues. Nonetheless, we consider that the amelioration of adverse remodeling in the treated groups supports the beneficial effect of the intracoronary infusion of pCSCs at both early and 7 days after AMI.

## Conclusions

In conclusion, the present study suggests that the intracoronary administration of allogeneic pCSCs represents a safe and clinically applicable ancillary therapy that alleviates myocardial dysfunction, especially when performed one week after the insult. It may have a timing-related effect, with early infusion improving angiogenesis, and later infusion having a greater effect on cardiac function as measured with cMR during this limited follow-up time. However, and despite the absence of MACEs during injection or cardiac deaths in any group, safety concerns along with a greater improvement of cardiac function in this experimental setting recommend the use of the 1week time point for the intracoronary injection of allogeneic CSCs.

## Additional files

Additional file 1:
**Evaluation of porcine CSC differentiation capabilities to cardiac lineages.**
Additional file 2:
**Immuno-cytochemistry analysis of porcine Cardiac Stem Cells after differentiation into the three major cardiac lineages.**
