# Supplementary material for: Delayed administration of allogeneic cardiac stem cell therapy for acute myocardial infarction could ameliorate adverse remodeling: experimental study in swine
Source: J Transl Med. 2015 May 12;13:156. doi: 10.1186/s12967-015-0512-2 (PMC4458045; doi:10.1186/s12967-015-0512-2)
Supplement: Additional file 1: — Evaluation of porcine CSC differentiation capabilities to cardiac lineages. [file 12967_2015_512_MOESM1_ESM.docx]

Delayed administration of allogeneic Cardiac Stem Cell therapy for acute myocardial infarction could ameliorate adverse remodelling. Experimental study in swine.

Veronica Crisostomo^a^, Claudia Baez-Diaz^a^, Juan Maestre^a^, Monica Garcia-Lindo^a^, Fei Sun^a^, Javier G. Casado^a^, Rebeca Blazquez^a^, Jose L. Abad^b^, Itziar Palacios^b^, Luis Rodriguez-Borlado^b^, Francisco M. Sanchez-Margallo^a^.

*a.- Jesús Usón Minimally Invasive Surgery Centre*

Carretera N-521, km 41.8.

10071 – Cáceres, Spain.

*b.- Coretherapix*

Santiago Grisolía, nº 2 (Parque Científico de Madrid)

28760 Tres Cantos (Madrid, Spain)

**Supplementary materials and methods**

**Immunocytochemical:** To evaluate porcine CSC differentiation capabilities to cardiac lineages we followed the protocol previously described by Smith et al (Nature Protocols 2014: 9(7): 1662-1681), pCSC isolated from heart samples by cKit immunoselection and *in vitro* expanded were plated on 0.1% pre-coated plates and cultured for 3 days. Cells were then trypsinized and seeded on Ultra-Low attachment plates for 3 additional days to form cardiospheres. Once formed, cardiospheres were collected and seeded onto plates covered with laminin in differentiation media (α-MEM, FBS 2%), plus dexamethasone (1 µM), β-glycerolphosphate (10 nM), and ascorbic acid (50 µg/mL). During four days medium was supplemented with TGF-β1 (5 ng/mL), BMP-2 (10 ng/mL) and BMP-4 (10 ng/mL). After that, the supplements (TGF-β1, BMP-2, BMP-4) were replaced by DKK-1 (0.15 µg/mL) and cells were cultured in cover slides during 30.days. Cells were then harvested for immunofluorescence staining. Briefly, cells were fixed with 4% Paraformaldehyde (PFA), blocked with 1% Bovine Serum Albumin (BSA) for 1h at RT, and incubated with the corresponding antibody (see supplementary table). After 1h of incubation at RT, culture slides were washed in PBS (2 x 5’ each) and once in 0.1% Tween20 PBS (5’) and incubated with the corresponding secondary antibody. Cell nuclei were stained with 4´-6-diamidino-2-phenylindole (DAPI-Vectorshield) and stored at 4ºC until confocal microscope analysis.

**Results:**

The results show that porcine CSC have the ability to express high levels of Smooth Muscle Actin when cultured in differentiation medium and to induce the up-regulation of the endothelial cell marker von Villebrand Factor (vWF). Regarding the differentiation to mature cardiomyocytes we were not able to obtain fully mature beating cardiomyocytes but we observed the up-regulation of cardiomyocyte markers. The junctional protein Connexin 43 expression was induced and detected around the edges of the cells after differentiation (Fig X ). Staining of the contractile troponin I protein showed a striated pattern as it has been described in mature cardiomyocytes. The induction of cardiomyogenic marker such as Sarcomeric Actinin, did not show the expected distribution in the Z-discs.

These data show that porcine CSC are multipotent and have the ability to differentiate to the several cardiac lineages.

Supplementary table. Antibodies used in the study

| Lineage | Antibody | Cat Number |
| --- | --- | --- |
| **Cardiomyocytes** | α- Cardiac Troponin I | sc-15368 (Santa Cruz) |
|  | α- Connexin 43 | C8093 (Sigma) |
|  | α- Sarcomeric Actinin | A2172 (Sigma) |
| **Smooth Muscle cells** | α- Smooth Muscle Actin | A2547 (Sigma) |
| **Endothelial cells** | α- von Willebrand Factor | A0082 (Dako) |
