# Supplementary figures and images for: Delayed administration of allogeneic cardiac stem cell therapy for acute myocardial infarction could ameliorate adverse remodeling: experimental study in swine
Source: J Transl Med. 2015 May 12;13:156. doi: 10.1186/s12967-015-0512-2 (PMC4458045; doi:10.1186/s12967-015-0512-2)

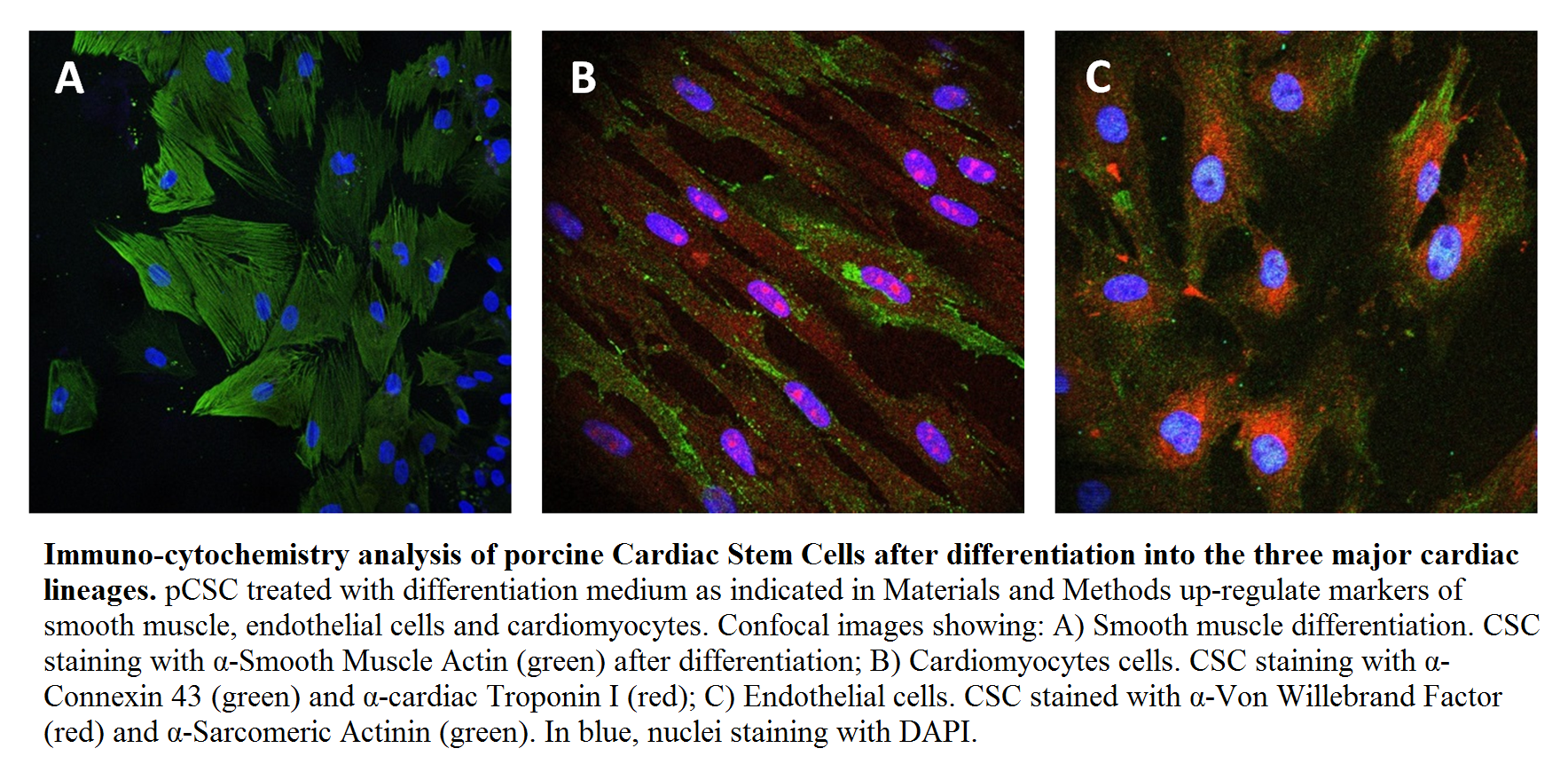

Supplement: Additional file 2: — Immuno-cytochemistry analysis of porcine Cardiac Stem Cells after differentiation into the three major cardiac lineages. [file 12967_2015_512_MOESM2_ESM.tiff]
